# Clinical picture and laboratory result of diabetes mellitus type-1

**DOI:** 10.1186/1687-9856-2013-S1-P29

**Published:** 2013-10-03

**Authors:** Harjoedi Adji Tjahjono

**Affiliations:** 1 Saiful Anwar Hospital, Malang, Indonesia

## 

The most commonly type 1 diabetes mellitus caused by destruction of β cell pancreatic leading to inadequate insulin production. Clinical and laboratory features at the times of diagnosis have been widely studied. We reviewed the clinical appearance of Type 1 Diabetes Mellitus in patients were diagnosed in the Pediatric Department of Saiful Anwar Hospital between 2005 and 2009.

The study has been conducted at Pediatric Department of Saiful Anwar Hospital between 2005 and 2009. A. descriptive study of clinical appearance of Type 1 Diabetes Mellitus of 27 patients, 1-14 years of age.

## Result

the incidence rate of Type 1 Diabetes Mellitus in our clinic during the period from 2005 to 2009 was 0.0034%. There were thirty Type 1 Diabetes Mellitus patients with duration of illness of more than 2 years, with a male to female ratio of 1: 1.7. Most of these patients had no diabetic family history and had moderate malnourished nutritional status. The average frequency of blood glucose home monitoring was less than ideal. Twenty-three out of the 27 patients were fully controlled metabolically; however, these patients still have polyuria, polydipsia, and polyphagia.

All of patients type-1 diabetes mellitus have polyuria, polydipsia, polyphagia and weight loss. The majority of age was 5-10 years and female. Most patients had no history of Diabetes mellitus. Most patients hospitalized with Diabetes Keto Acidosis, moderate malnourished, and blood glucose level more than 300 mg/dl.

